# OsMADS57 together with OsTB1 coordinates transcription of its target *OsWRKY94* and *D14* to switch its organogenesis to defense for cold adaptation in rice

**DOI:** 10.1111/nph.14977

**Published:** 2018-01-24

**Authors:** Liping Chen, Yuan Zhao, Shujuan Xu, Zeyong Zhang, Yunyuan Xu, Jingyu Zhang, Kang Chong

**Affiliations:** ^1^ Key Laboratory of Plant Molecular Physiology Institute of Botany Chinese Academy of Sciences Beijing 100093 China; ^2^ University of Chinese Academy of Sciences Beijing 100049 China; ^3^ National Center for Plant Gene Research Beijing 100093 China

**Keywords:** cold tolerance, D14, gene network, organogenesis, OsMADS57, rice (*Oryza sativa*), trade‐off, WRKY94

## Abstract

- Plants modify their development to adapt to their environment, protecting themselves from detrimental conditions such as chilling stress by triggering a variety of signaling pathways; however, little is known about how plants coordinate developmental patterns and stress responses at the molecular level.
- Here, we demonstrate that interacting transcription factors OsMADS57 and OsTB1 directly target the defense gene *OsWRKY94* and the organogenesis gene *D14* to trade off the functions controlling/moderating rice tolerance to cold.
- Overexpression of *OsMADS57* maintains rice tiller growth under chilling stress. OsMADS57 binds directly to the promoter of *OsWRKY94*, activating its transcription for the cold stress response, while suppressing its activity under normal temperatures. In addition, *OsWRKY94* was directly targeted and suppressed by OsTB1 under both normal and chilling temperatures. However, *D14* transcription was directly promoted by OsMADS57 for suppressing tillering under the chilling treatment, whereas *D14* was repressed for enhancing tillering under normal condition.We demonstrated that OsMADS57 and OsTB1 conversely affect rice chilling tolerance via targeting *OsWRKY94*.
- Our findings highlight a molecular genetic mechanism coordinating organogenesis and chilling tolerance in rice, which supports and extends recent work suggesting that chilling stress environments influence organ differentiation.

## Introduction

Rice (*Oryza sativa*) is a major staple crop for over half of the world's population. One of the key issues in rice production is its limited adaptability to local environmental stresses such as chilling, as it evolved in tropic and temperate regions (Kovach *et al*., 2007; Sang, 2011). Improvement of plant chilling tolerance could enable the expansion of rice cultivation to more northerly latitudes.

Chilling tolerance is a complex agronomic trait controlled by genetic networks and signal transduction pathways (Thomashow, 1999; Yamaguchi‐Shinozaki & Shinozaki, 2006; Zhu *et al*., 2007; Zhang *et al*., 2014; Zhao *et al*., 2015). Interactions between COLD1 (CHILLING TOLERANCE DIVERGENCE 1) and RGA1 (rice G‐protein α subunit 1) enable cold signaling to confer chilling tolerance in *japonica* rice (Ma *et al*., 2015; Shi & Yang, 2015) through the subsequent calcium signaling triggered in response to downstream stress response networks of C‐repeat‐binding factor (CBF) transcription factors(Sangwan *et al*., 2002; Knight & Knight, 2012; Zhu, 2016). However, less is known about how plants coordinate the stress response with developmental patterning to adapt to their environment over the longer term.

Developmental plasticity enables plants to respond to abnormal ambient temperatures by reprogramming their gene expression to adapt their architecture. Cold temperatures can disrupt the intrinsic signal network in the shoot apical meristem (SAM), and set a dormancy cycling at the SAM to enhance stress tolerance (van der Schoot & Rinne, 2011). The protection of root stem cell niche forms a sacrifice‐for‐survival mechanism from chilling stress (Hong *et al*., 2017). This altered development in response to temperature is crucially important for the maintenance of meristematic activity and the proper organization of differentiated cells (Komaki & Sugimoto, 2012). Under chilling treatment, several specific genes involved in the cell cycle are activated by transcription factors such as OsMYB3R‐2 to keep the cell mitotic for their tolerance (Ma *et al*., 2009). It suggests that the capability of maintaining cell behavior and activity is a resource to enhance survival and growth during and/or after chilling stress.

Shoot branching is controlled by the formation and subsequent outgrowth of axillary buds in the axils of the leaf primordia. *LAX PANICLE 1* (*LAX1*) and *MONOCULM1* (*MOC1*) control axillary bud initiation(Komatsu *et al*., 2003; Li *et al*., 2003).The DWARF genes, including *D3*,* D10*,* D14*,* D17*,* D27* and *D53*, play critical roles in the strigolactone biosynthesis and signaling pathways during axillary bud outgrowth, which is required for tillering in rice(Ishikawa *et al*., 2005; Arite *et al*., 2007, 2009; Yan *et al*., 2007; Jiang *et al*., 2013).*OsTB1/FC1* acts downstream of the *DWARF* genes to repress the outgrowth of axillary buds in rice (Takeda *et al*., 2003; Minakuchi *et al*., 2010). OsTB1 interacts with OsMADS57 to reduce its inhibitory impact on *D14*, a gene encoding strigolactone receptor that controls the organogenesis of tillers(Zhao *et al*., 2013; Yao *et al*., 2016), enabling it to regulate axillary bud initiation(Guo *et al*., 2013). Axillary buds are indeterminate structures that can be developmentally controlled in response to endogenous or environmental cues (Domagalska & Leyser, 2011; Janssen *et al*., 2014).However, the regulatory network influencing axillary bud development in response to stress is unknown.

In the present study, a network with a core OsMADS57 was identified to trade off chilling tolerance and axillary bud for adaptation to cold environment. We present evidence that OsMADS57 represents a new class of regulator, functioning in conjunction with OsTB1 to optimize chilling stress tolerance in rice seedlings. Our investigation revealed that OsMADS57 and OsTB1 can both directly target a transcription factor *OsWRKY94* and axillary bud regulated gene *D14* for adaptation to cold, thus providing evidence that OsMADS57 acts as a molecular link between the developmental response and chilling stress tolerance in rice.

## Materials and Methods

### Plant material and growth conditions

The *osmads57‐1* and *d14* mutants, as well as the *OsMADS57* overexpression (*OsMAD57*‐OE) and antisense (*OsMADS57*‐AS) lines, were described previously (Guo *et al*., 2013). The T‐DNA insertion mutant lines PFG_3A‐15619.R (*osmads57‐2*) and PFG_3A‐13305.R (*oswrky94‐1*) in the *Oryza sativa* L. ssp. *japonica* cv Dongjin background were obtained from RiceGE, the Rice Functional Genomics Express Database of Korea. The *ostb1* mutants were a gift from Professor Qian Qian, China National Rice Research Institute.

### Chilling stress treatment

Rice plants were grown in a temperature‐controlled glasshouse with 30°C : 28°C, day : night cycles in Kimura B nutrient solution (Kato‐Noguchi & Ino, 2005) for 2 wk, then placed in a 4°C circulating water bath for the chilling treatment for various periods of time. The evaluation of chilling tolerance with survival rate was performed as described by Ma *et al*. (2015).After the chilling treatment, the plants were frozen in liquid nitrogen for further analyses.

### Gene expression analysis

Plant total RNA was isolated using a TRIzol RNA Extraction Kit (Invitrogen) and treated with RNase‐free DNase I (MBI Fermentas, Waltham, MA, USA). A 2‐μg aliquot of total RNA was used to synthesize cDNA using AMV Reverse Transcriptase (Promega). The cDNA was diluted 1 : 50 into 15 μl SYBR Green quantitative PCR Master Mix (Toyobo, Osaka, Japan), according to the manufacturer's instructions. Quantitative reverse transcription polymerase chain reaction (RT‐PCR) was performed on a Mx3000P instrument (Stratagene, La Jolla, CA, USA). The gene expression levels were normalized to that of *UBIQUITIN* (*UBQ*). Each experiment included three technical replicates and at least three biological replicates. The values represent means ± SD of three technical replicates. Primers used are listed in Supporting Information Table S1.

### Yeast one‐hybrid assays

Yeast one‐hybrid assays were used to check the binding of OsMADS57 and OsTB1 to the promoter of *OsWRKY94*. The coding sequences of *OsMADS57* and *OsTB1* were independently cloned into the *Eco*RI–*Xho*I site of the pJG4‐5 vector as prey. The effectors contained the GAL4‐activation domain. DNA fragments corresponding to the promoters of *OsWRKY94* and *D14* were independently cloned into the pLacZi plasmid as bait. Primers used for cloning are listed in Table S1. These constructs were transformed into the *Saccharomyces cerevisiae* strain EGY48. The transformed yeast was selected on a synthetic complete medium lacking Ura and Leu.

### Protoplast transformation and transient expression assays

In order to assess the transient expression of *OsWRKY94* driven by OsMADS57 and OsTB1, the cDNAs of full‐length *OsMADS57*,* OsMADS57N* and *OsTB1* were fused into the pBI221 vector driven by the *35S* promoter as effectors. The reporter plasmids *OsWRKY94p::LUC* and *D14p::LUC* were generated, and the *35S::GUS* plasmid was used as a normalization control (LUC, luciferase; GUS, β‐glucuronidase). A transcriptional activity assay was carried out in the transiently transformed *Arabidopsis thaliana* protoplast system (Lin *et al*., 2007). The values represent means ± SD of three technical replicates. Co‐transformation of OsMADS57 and OsTB1 was performed to identify the effect of OsTB1 in the transient assay. As a control, OsTB1 (OsTB1^V159QWL162L163^) was replaced with OsTB1m (OsTB1^*A*159QW*A*162*A*163^) to disturb its interaction with OsMADS57 (Heery *et al*., 1997; Guo *et al*., 2013). After transformation, the *Arabidopsis* protoplasts were incubated at 22°C for 18 h followed by a treatment of 25°C or 4°C for an additional 30 min. Relative LUC activity (LUC/GUS) was calculated to determine *OsWRKY94* promoter activity (Lin *et al*., 2007).

For the OsWRKY94 transcriptional activity assay, a GAL4 binding domain (BD)‐OsWRKY94 fusion protein was generated, which can bind to the GAL4 DNA‐binding sites of the *GUS* reporter. The transcription suppressor HOS15 and a transcription activator, ARF5M, were used as the controls (Zhu *et al*., 2008). A *GUS* reporter containing four upstream GAL4 DNA‐binding sites (*GAL4*(4X)‐ D1‐3(4X)‐ *GUS*) and the *35S::LUC* internal control were co‐transformed with *GAL4 BD‐OsWRKY94* into *Arabidopsis* protoplasts. After cell lysis, a 5‐μl extract was mixed with 45 μl LUC Assay Substrate (Promega) to determine LUC activity. For the GUS activity assay, a 5‐μl extract was incubated with 45 μl 4‐methylumbelliferyl β‐d‐glucuronide assay buffer (50 mM sodium phosphate pH 7.0, 1 mM 4‐methylumbelliferyl β‐d‐glucuronide, 10 mM EDTA, 10 mM β‐mercaptoethanol, 0.1% sarkosyl and 0.1% Triton X‐100) at 37°C for 30 min, after which the reaction was terminated by adding 950 μl 0.2 M Na_2_CO_3_. The GUS : LUC ratio was calculated as the relative reporter expression level.

### Electrophoretic mobility shift assays

GST‐OsMADS57 and GST‐OsTB1 recombinant proteins were expressed in the *Escherichia coli* BL21 (DE3) strain and purified using Glutathione Sepharose 4B beads (GE Healthcare, Stockholm, Sweden) (GST, Glutathione S‐transferase). An electrophoretic mobility shift assay (EMSA) was performed using a LightShift Chemiluminescent EMSA Kit (Pierce, Waltham, MA, USA), according to the manufacturer's protocol with some previously described modifications (Ma *et al*., 2009). Oligonucleotides complementary to different motifs of the *OsWRKY94* promoter were synthesized, annealed, and labeled using a Biotin 3′ End DNA Labeling Kit (Pierce). The oligonucleotide sequences are shown in Table S1.

### Chromatin immunoprecipitation

Chromatin immunoprecipitation (ChIP) was performed as described previously (Bowler *et al*., 2004; Liu *et al*., 2014). The FLAG‐OsMADS57 line and the wild‐type (WT) ZH10 (*Oryza sativa* L. ssp. *japonica* cv Zhonghua 10), were grown for 2 wk and used to carry out the ChIP assay. Root‐dislodged seedlings (2 g) were cross‐linked for 20 min with 1% formaldehyde under a vacuum. Chromatin complexes were isolated and fragmented as described previously (Liu *et al*., 2014). An anti‐FLAG polyclonal antibody (Abcam, Cambridge, UK) and Protein A agarose/salmon sperm DNA (Millipore, Darmstadt, Germany) were used for immunoprecipitation. After reverse cross‐linking and protein digestion, the DNA products were analyzed using quantitative RT‐PCR. The enrichment was calculated as the ratio of FLAG‐OsMADS57 to ZH10. Values are the means ± SD of three independent experiments. The primers used in the ChIP assays are listed in Table S1.

### Surface plasmon resonance analysis

All experiments were performed using a Biacore 3000 instrument and streptavadin‐coated biosensor chips (SA‐Chip; GE Healthcare) (Henriksson‐Peltola *et al*., 2007; Wang *et al*., 2015). All buffers were freshly prepared, filtered using 0.22‐μm syringe filters and de‐gassed. The instrument was first primed three times with reaction buffer (10 mM Hepes PH 7.9, 50 mM KCl, 1 mM EDTA, 0.5 mM DTT, 5 mM MgCl_2_, 10% Glycerol, 0.005% surfactant P20) and flow cell 1 (FC1) was used as the reference flow cell, which was unmodified and lacked the oligonucleotide ligand. Flow cell 2 (FC2) was used for the immobilization of the oligonucleotide. It was conditioned with three consecutive 1‐min injections of 50 mM NaOH in 1 M NaCl, in accordance with the manufacturer's instructions. The biotin‐labeled oligonucleotide (the same as was used in the EMSA) was then injected over a 1‐min period at a flow rate of 5 μl min^−1^, followed by the extraclean feature. Oligonucleotide immobilization levels of 200–300 RU were routinely observed under these conditions. Protein‐DNA binding assays were performed in the reaction buffer at the relatively high flow rate of 10 μl min^−1^ to avoid or minimize any mass‐transport limitation effects. Protein solutions were injected for 120 s followed by a dissociation in reaction buffer for 280 s. At the end of the dissociation period, the sensor chip was regenerated to remove any remaining bound material by injecting reaction buffer, containing 50 mM NaOH and 1 M NaCl, at 30 μl min^−1^ for 30 s.

## Results

### *OsMADS57* is required for chilling tolerance in rice

**Figure 1:**
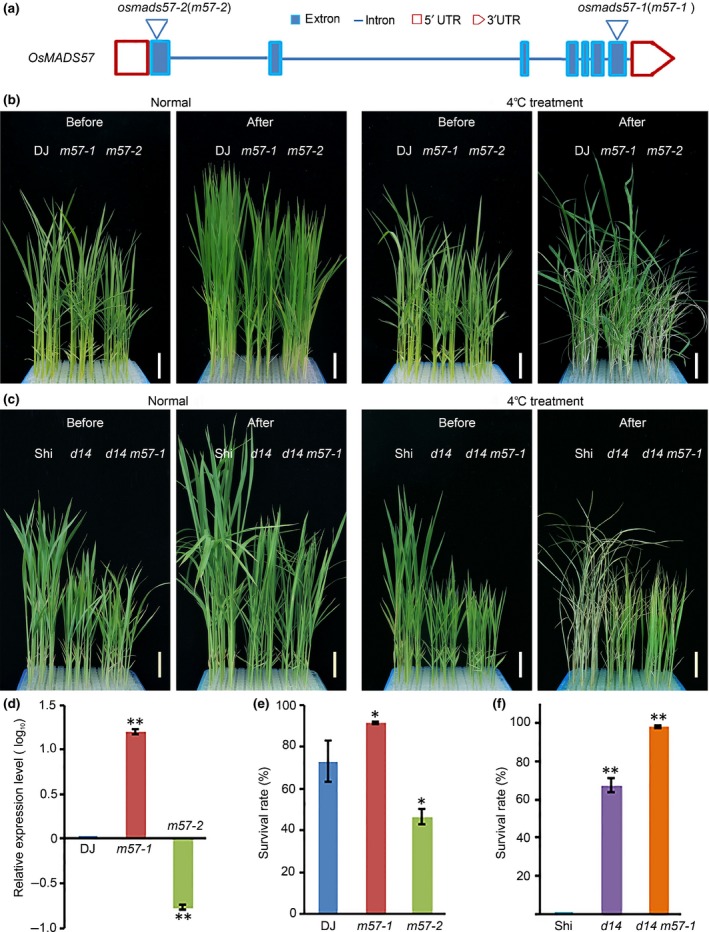
*OsMADS57* is required for chilling tolerance. (a) Diagram of *OsMADS57* gene structure. The triangles represent the T‐DNA insertion sites in *osmads57‐1* (*m57‐1*) and *osmads57‐2* (*m57‐2*) mutants. (b) Chilling tolerance phenotype of the gain‐of‐function mutant *osmads57‐1* (*m57‐1*), the loss‐of‐function mutant *osmads57‐2* (*m57‐2*) and the wild‐type (WT) Dongjin (DJ,* Oryza sativa* ssp. *japonica* cv Dongjin). Seedlings were incubated at 4°C for 84 h, then transferred back to the normal condition for recovery. Bars, 2.5 cm. (c) Chilling tolerance phenotype of the loss‐of‐function mutant *d14*, the double mutant *d14 osmads57‐1* (*d14 m57‐1*) and the WT Shiokari (Shi, *Oryza sativa* ssp. *japonica* cv Shiokari). Seedlings were incubated at 4°C for 96 h, then transferred back to the normal condition for recovery. Bars, 2.5 cm. (d) Expression analysis of *OsMADS57* in *osmads57‐2* (*m57‐2*) using quantitative reverse transcription polymerase chain reaction. Values are expressed as the mean ± SD,* n *=* *3 (three technical replicates per biological repeat). **, *P *<* *0.01 (Student's *t*‐test). (e) Survival rate (percentage of seedlings surviving) of seedlings from (a) following the chilling treatment. Values are expressed as mean ± SD,* n *=* *3, *, *P *<* *0.05 (Student's *t*‐test). (f) Survival rate of seedlings from (b) following the chilling treatment. Values are expressed as mean ± SD,* n *=* *3, **, *P *<* *0.01 (Student's *t*‐test).

The gain‐of‐function rice mutant *osmads57‐1*, in which the T‐DNA was inserted in the 3′terminus of *OsMADS57*, exhibits an enhanced outgrowth of axillary buds that increases its number of tillers (Guo *et al*., 2013). The *osmads57‐2* mutant line (PFG_3A‐15619.R)(Jeong *et al*., 2002) contains a T‐DNA insertion in the first exon of *OsMADS57*, 61 bp from the transcriptional initiation site, which results in a loss of the full‐length transcript(Figs 1a,d, S1a–d). Therefore, it is a knockdown mutant on the genome.

At the transcription level, *OsMADS57* expression was significantly induced during abiotic stresses such as salt, drought, abscisic acid and chilling (Fig. S1e)(Arora *et al*., 2007). To examine the genetic function of *OsMADS57* in response to chilling, seedlings of the *osmads57* mutants were exposed to chilling temperatures (4°C) and subsequently returned to normal conditions (30°C : 28°C, day : night) for recovery. Rice seedlings with chilling tolerance were defined as those that could differentiate new leaves or continue leaf growth during the recovery treatment (Ma *et al*., 2015). After 7 d of recovery from the 84‐h chilling treatment, 91% of the *osmads57‐1* seedlings survived, in contrast to 73% of WT Dongjin (DJ, *Oryza sativa* L. ssp. *japonica* cv Dongjin). Only 46% of *osmads57‐2* plants survived after the chilling treatment (Fig. 1b,e). The data suggest that *OsMADS57* is required for chilling tolerance in rice.

OsMADS57 negatively regulates *D14* transcription expression, affecting tiller production (Guo *et al*., 2013). To assess the possibility of *D14* being involved in the chilling response genetic network, the *d14* mutant was exposed to a chilling treatment.The *d14* seedlings exhibited an obviously more tolerant phenotype (67% of the seedlings survived) than the WT Shiokari (Shi, *Oryza sativa* L. ssp. *japonica* cv Shiokari)(none of the seedlings survived), whereas the double mutant *d14 osmads57‐1* showed a higher survival rate (98% of the seedlings survived) than the *d14* single mutant (Fig. 1c,f). This suggests that the regulation of *D14* by OsMADS57 might be involved in the chilling stress response.

During the recovery period following the chilling treatment, the leaves of the *osmads57‐2* mutant seedlings withered, which was particularly noticeable in the younger leaves (Fig. S2a). This withering was less pronounced in the WT, whereas the leaves of the *osmads57‐1* plants were green and grew well, with new leaves being formed. After > 14 d of recovery, the *osmads57‐1* mutants had notable new tiller growth, in contrast to the WT and the *osmads57‐2* mutants (Fig. S2b,c, Methods S1).The mitotic index in the root apical meristem did not differ significantly between the WT and the *osmads57* mutants when grown in normal temperatures. By contrast, when subjected to chilling conditions, the mitotic index of the gain‐of‐function mutant *osmads57‐1* was higher than that of the WT, whereas that of the loss‐of‐function mutant *osmads57‐2* was lower than that of the WT (Fig. S3a,b).This suggests that OsMADS57 could maintain cell division under chilling stress.

The overexpression line of *OsMADS57* (*OsMAD57‐*OE) (Guo *et al*., 2013) showed a higher survival rate (70%) than its WT, ZH10 (37%), after the chilling treatment. By contrast, the antisense line (*OsMADS57‐*AS) was sensitive to chilling stress, and its survival rate (25%) was significantly lower than ZH10 (Fig. S4a,b). After > 14 d of recovery from the chilling treatment, the *OsMADS57*‐OE plants showed obvious tiller outgrowth, in contrast to the WT, whereas the *OsMADS57‐*AS plants that survived showed no obvious difference in tiller number compared with the WT (Fig. S4c). This result implies that OsMADS57 may play a role in both tillering and cold tolerance.

### OsMADS57 directly activates *OsWRKY94* expression in response to chilling

**Figure 2:**
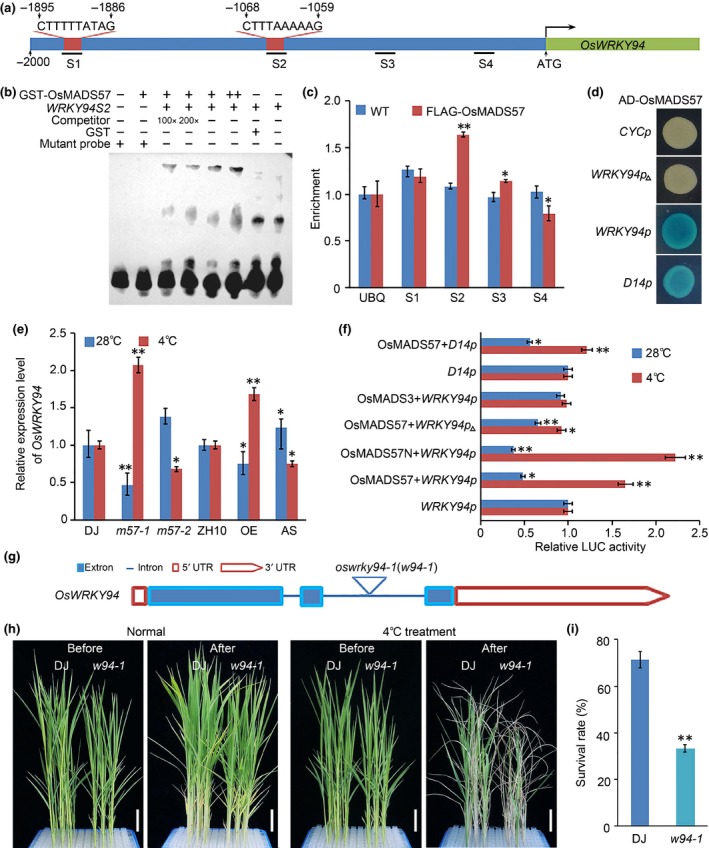
OsMADS57 directly upregulates *OsWRKY94* expression during chilling treatment. (a) Schematic of the *OsWRKY94* promoter showing the CArGboxes in red (S1 and S2). The S1 sequence is CTTTTTATAG and the S2 sequence is CTTTAAAAAG. Black lines S1–S4 indicate the sequences tested in the chromatin immunoprecipitation (ChIP) assays. (b) *In vitro* electrophoretic mobility shift assay (EMSA) showing that the GST‐OsMADS57 fusion protein binds to the *OsWRKY94* promoter (*WRKY94S2*). The oligos were synthesized by fusing three copies of the S2 motif and their flanking sequence. The mutant probe (AGGGCCCCCT) served as the negative control. (c) ChIP to measure OsMADS57 occupancy at the *OsWRKY94* promoter *in vivo*. The DNA isolated using ChIP was analyzed using quantitative PCR. Values are expressed as mean ± SD,* n *=* *3. The *UBIQUITIN* (*UBQ*) promoter was used as a negative control.*, *P *<* *0.05; **, *P *<* *0.01 (Student's *t*‐test). (d) Yeast one‐hybrid analysis. *D14p* represents the positive control. *WRKY94p*_▵_ is the truncated promoter of *OsWRKY94* in which the CTTTAAAAAG sequence is absent. *CYCp* is the negative control of the blank vector. (e) *OsWRKY94* expression in response to the chilling treatment in lines expressing various *OsMADS57* constructs. Values are expressed as mean ± SD,* n *=* *3. *, *P *<* *0.05;**, *P *<* *0.01 (Student's *t*‐test). (f) Transient transcriptional assay of *OsWRKY94* driven by OsMADS57 in *Arabidopsis* protoplasts.*, *P *<* *0.05; **, *P *<* *0.01 (Student's *t*‐test). (g) Diagram of *OsWRKY94* gene structure. The triangles represent the T‐DNA insertion sites in *oswrky94‐1* (*w94‐1*) mutant. (h) Phenotype and (i) survival rate of *oswrky94‐1* (*w94‐1*) and the wild‐type, Dongjin (DJ,* Oryza sativa* ssp.*japonica* cv Dongjin) following 84 h of the chilling treatment. Values are expressed as mean ± SD,* n *=* *3, **, *P *<* *0.01 (Student's *t*‐test). Bars, 2.5 cm.

A previous microarray assay of the *osmads57‐1* mutant revealed the candidate targets of OsMADS57 (Guo *et al*., 2013). These include *WRKY* genes predicted to function in plant developmental processes and in the response to abiotic stresses. By using the putative OsMADS57‐binding motif to search all of the *WRKY* gene promoters in rice, two potential OsMADS57‐binding CArGboxes, site 1 [CTTTTTATAG] and site 2[CTTTAAAAAG], were identified 1895–1886 bp and 1068–1059 bp upstream of the *OsWRKY94* transcription start site, respectively (Fig. 2a). Site 2 comprised the same sequence as the OsMADS57‐binding motif in *D14*. To test whether OsMADS57 binds to *OsWRKY94*, an EMSA was used. As shown in Fig. 2(b), a clear OsMADS57‐dependent mobility shift was identified, and incubation with the OsMADS57 protein caused strong mobility shift bands in the probes for site 2 (lanes3–6) as compared with the probe alone (lane 8), or the GST protein alone (lane7). The unlabeled *WRKY94S2* probe competed for the binding of labeled *WRKY94S2* to MADS57 protein. As a negative control, the mutant probe did not show any shifted band when incubated with labeled *WRKY94S2* probe (Fig. 2b).To further determine whether OsMADS57 directly associates with *OsWRKY94 in vivo*, a ChIP assay was performed using transgenic FLAG‐OsMADS57 lines. An assay using the FLAG antibody showed that the ‘S2’ region of the *OsWRKY94* promoter, involving the site 2 CArG motif, was enriched in FLAG‐OsMADS57 compared with the WT, ZH10 (Fig. 2c). By contrast, there were no obvious enrichment for regions ‘S1’ and ‘S4’.The activities of OsMADS57 on *OsWRKY94* also were tested in yeast one‐hybrid assays via the expression of the reporter gene *LacZ* driven by the *OsWRKY94* promoter‐*Pcyc1* (*WRKY94p*). The strains treated with OsMADS57 to induce *OsWRKY94* expression grew well and were blue (Fig. 2d). By contrast, yeast cells treated with OsMADS57, but lacking the *OsWRKY94* promoter, did not turn blue. *D14* was used as a positive control for the CArG box, and yeast cells treated with OsMADS57 to drive *D14* expression turned blue. *WRKY94p*_▵_, a truncated form of the *OsWRKY94* promoter that lacked the CArG box, was used as a negative control. Thus, OsMADS57 targets the CArG *cis*‐elementat site 2, which was present in the promoter of *OsWRKY94*. Our results suggested that OsMADS57 could directly target *OsWRKY94*.

Transcription of *OsWRKY94* was upregulated in either *osmads57‐1* or *OsMADS57*‐OE seedlings subjected to the chilling treatment, but was repressed under normal growth conditions (Fig. 2e). Conversely, *OsWRKY94* expression was downregulated in either *osmads57‐2* or *OsMADS57*‐AS plants following chilling. This suggested that the expression of *OsWRKY94* is positively regulated by OsMADS57 under chilling stress *in planta*.

In order to confirm the effect of OsMADS57 on *OsWRKY94* transcription, we carried out a transient expression assay in *Arabidopsis* mesophyll protoplasts. The data showed that OsMADS57 repressed the expression of the *LUC* reporter gene driven by the *OsWRKY94* promoter under normal temperatures (25°C) (Fig. 2f). When the transformed protoplasts were treated with low temperatures (4°C) for 30 min, the expression of *OsWRKY94* was strongly activated by OsMADS57 rather than being repressed, and to a greater extent than its repression in normal growth conditions. The activation of *OsWRKY94* by OsMADS57 was diminished by the use of a truncated promoter missing the site 2 CArG box. The activity of the OsMADS57 N‐terminal peptide (170 aminoacid residues; OsMADS57N) was analyzed in the assay. OsMADS57N repressed *OsWRKY94* expression under normal conditions, and activated *OsWRKY94* expression under the chilling stimulus. It is consistent with our previous study that truncated OsMADS57 protein lacking the C‐terminus is sufficient for its function (Guo *et al*., 2013). *D14*, the positive control, was repressed by OsMADS57 under normal conditions but the activity showed little activation under chilling stress. OsMADS3, the negative control, showed no significant change in *LUC* expression either under low temperatures or normal growth conditions. The data suggest that OsMADS57 can act on *OsWRKY94* either as a repressor or as an activator in a temperature‐dependent manner.

### OsWRKY94 positively regulates chilling tolerance

The *oswrky94‐1* mutant contains a T‐DNA insertion 946 bp from the transcription initiation site of *OsWRKY94*, in the second intron, identified by genome sequencing (Figs 2g, S5a,b)(Jeong *et al*., 2002).This T‐DNA insertion results in the lack of the conserved WRKY domain for *OsWRKY94*. A homozygous *oswrky94‐1* mutant was identified using specific primers from rice GE(Fig. S5c), and transcription expression assays using semiquantitative and quantitative PCRs showed that *oswrky94‐1* is a knockdown mutant in the genome (Fig. S5d,e). A phylogenetic analysis revealed that OsWRKY94 shares a higher sequence similarity with OsWRKY121 than with other homologs of WRKY proteins in rice and *Arabidopsis* (Fig. S5f, Methods S1).

Clear differences in survival rate were shown between *oswrky94‐1* and its WT, DJ, after the chilling treatment (Fig. 2h). After a 7‐d recovery from the chilling treatment, 33% of *oswrky94‐1* seedlings had survived, in contrast to 71% of the WTplants (Fig. 2i), suggesting that *OsWRKY94* is involved in chilling tolerance in rice.

**Figure 3:**
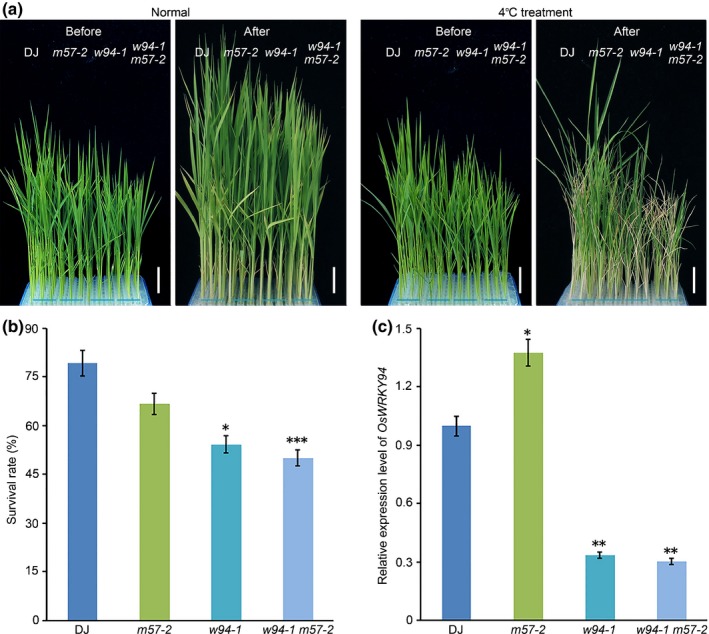
The *oswrky94‐1 osmads57‐2* (*w94‐1 m57‐2*) double mutant is sensitive to chilling stress. (a) Phenotype of *oswrky94‐1 osmads57‐2* (*w94‐1 m57‐2*) double mutant following 72 h of chilling treatment. Bars, 2.5 cm. (b) Survival rate of the *oswrky94‐1 osmads57‐2* (*w94‐1 m57‐2*) double mutant following the chilling treatment. Values are expressed as mean ± SD,* n *=* *3, *, *P *<* *0.05; ***, *P *<* *0.001. Student's *t*‐test. (c) *OsWRKY94* expression level in the *oswrky94‐1 osmads57‐2* (*w94‐1 m57‐2*) double mutant. Values are expressed as mean ± SD,* n *=* *3. *, *P *<* *0.05; **, *P *<* *0.01 (Student's *t*‐test).

The loss‐of‐function mutant *oswrky94‐1* and *osmads57‐2* showed a decreased chilling tolerance, and the double mutant *oswrky94‐1 osmads57‐2* exhibited a more significant sensitivity to chilling stress than either single mutant (Fig. 3a,b). Meanwhile, the expression level of *OsWRKY94* was more dramatically decreased in the *oswrky94‐1 osmads57‐2* double mutant than the *oswrky94‐1* single mutant under normal conditions (Fig. 3c), suggesting that *OsMADS57* and *OsWRKY94* may act in the same genetic pathway to regulate the chilling response.

Quantitative RT‐PCR assays showed that the transcript level of *OsWRKY94* was low in the leaves, but high in the root, shoot, culm, leaf sheath and spike (Fig. S6a). *OsWRKY94* expression was markedly induced by the chilling treatment (Fig. S6b). Transcriptional activity assays using protoplasts revealed that OsWRKY94 repressed 42% of the expression of the *GUS* gene which reports activity of OsWRKY94 compared with BD alone (set to 100%). The repressor HOS15 was used as a positive control, and reduced *GUS* expression to 11% of that with BD alone. The negative control, ARF5M, activated *GUS* expression by 2.6‐fold compared with BD only (Fig. S6c) (Zhu *et al*., 2008; Guo *et al*., 2013). Subcellular localization assays showed that OsWRKY94‐GFP fluorescence completely merged with H2B‐mCherry nuclear protein marker in rice protoplast (Fig. S6d, Methods S1). These results demonstrated that chilling‐induced *OsWRKY94* expression was required for its function in chilling tolerance.

Moreover, the transcription expression patterns of *WRKY* paralogs that could be putative targets of OsMADS57 were investigated. *OsWRKY8*,* OsWRKY50*,* OsWRKY62*,* OsWRKY71* and *OsWRKY108* all showed induced expression patterns in the WT following a chilling treatment. In *osmads57‐1, OsWRKY71* expression was upregulated. By contrast, expression of the *WRKY* paralogs also was induced in *osmads57‐2* in response to chilling (Fig. S6e). The *WRKY* paralogs may therefore display some functional redundancy.

### OsTB1 reduces chilling tolerance by directly downregulating *OsWRKY94*

**Figure 4:**
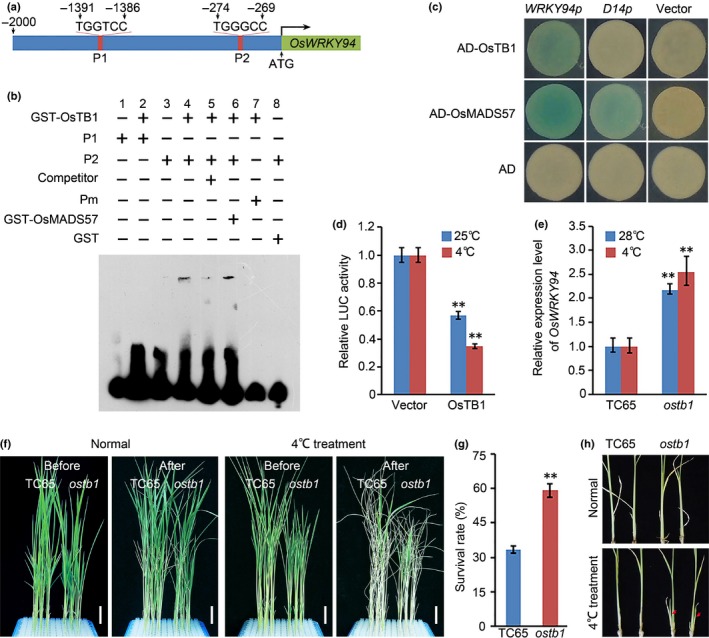
OsTB1 directly targets *OsWRKY94*. (a) Schematic of the*OsWRKY94* promoter showing the OsTB1 binding motif. The P1 sequence is TGGTCC and the P2 sequence is TGGGCC. (b) Electrophoretic mobility shift assay (EMSA). The GST protein and the mutant probe (Pm) served as the negative control. In Pm, TGGGCC was mutated to AAAAAA. Excessive amounts (×50) of unlabeled oligos were added as the competitors. (c) Yeast one‐hybrid analysis. The effectors contained the GAL4 activation domain. (d) Expression activity assay of *OsWRKY94* driven by OsTB1 in *Arabidopsis* protoplasts. **, *P *<* *0.01. Student's *t*‐test. (e) Quantitative reverse transcription polymerase chain reaction analyses of *OsWRKY94* transcript levels in seedlings of *ostb1* and the wild‐type, Taichung 65 (TC65, *Oryza sativa* L. ssp. *japonica* cv Taichung 65) following 24 h of chilling treatment. The expression levels in TC65 both before and after chilling treatment were defined as 1. Values are expressed as mean ± SD,* n *=* *3. **, *P *<* *0.01 (Student's *t*‐test). (f) Phenotype and (g) survival rate of *ostb1* following 84 h of chilling treatment. Values are expressed as mean ± SD,* n *=* *3, **, *P *<* *0.01 (Student's *t*‐test). Bars, 2.5 cm. (h) Chilling treatment increases tiller growth in *ostb1*. The arrows indicate the tillers that developed after the chilling treatment.

OsTB1 interacts with OsMADS57 to modulate tillering (Guo *et al*., 2013). The potential transcription factor binding motif of TCP (*TEOSINTE BRANCHED1* in *Zea mays*,* CYCLOIDEA* in *Antirrhinum majus*, and PROLIFERATING CELL FACTOR1 in *Oryza sativa*)(Aggarwal *et al*., 2010; Manassero *et al*., 2013)was searched in the *OsWRKY94* promoter, revealing two potential sites, site 1(TGGTCC) and site 2(TGGGCC), located 1386–1392 bp and 269–275 bp upstream of the initiation site of *OsWRKY94*, respectively(Fig. 4a). EMSA data revealed that site 2 (P2), but not site 1 (P1), was bound by the GST‐OsTB1 recombinant protein, whereas both sites were not bound by the GST protein alone (Fig. 4b). Addition of excess unlabeled probes reduced the binding. Addition of OsMADS57‐GST had no effect on the binding ability of OsTB1 to the promoter of *OsWRKY94*. A yeast one‐hybrid system assessing the activities of OsTB1 on the *OsWRKY94* promoter‐*Pcyc1* (*WRKY94p*) showed that the strains containing the *OsWRKY94* promoter grew well and became blue. By contrast, yeast cells treated with OsTB1 but lacking the *OsWRKY94* promoter did not turn blue. Under the same assay conditions, OsTB1 was found not to bind to *D14* (Fig. 4c).

A transcriptional regulation activity assay in protoplasts revealed that OsTB1 suppressed the expression of *LUC* driven by the *OsWRKY94* promoter under either normal temperature or the chilling treatment (Fig. 4d). By contrast, the chilling treatment enhanced directly the repression activity of OsTB1 on *OsWRKY94*. An expression pattern assay showed that *OsWRKY94* transcription was increased in the *ostb1* mutant compared with the WT. Chilling treatment enhanced the expression of *OsWRKY94* in *ostb1* compared with that in the WT (Fig. 4e). OsTB1 therefore directly suppresses *OsWRKY94* expression in response to change in the ambient temperature.

A phenotypic assay demonstrated that 59% of *ostb1* mutants were alive after the chilling treatment (Fig. 4f,g). By contrast, the WT Taichung 65 (TC65), had a 33% survival rate. After 14 d of recovery following the chilling treatment, the *ostb1* mutant showed obvious tiller outgrowth, which was not seen in the WT (Fig. 4h).

### OsMADS57 interacts with OsTB1 to coordinate rice growth and chilling tolerance

**Figure 5:**
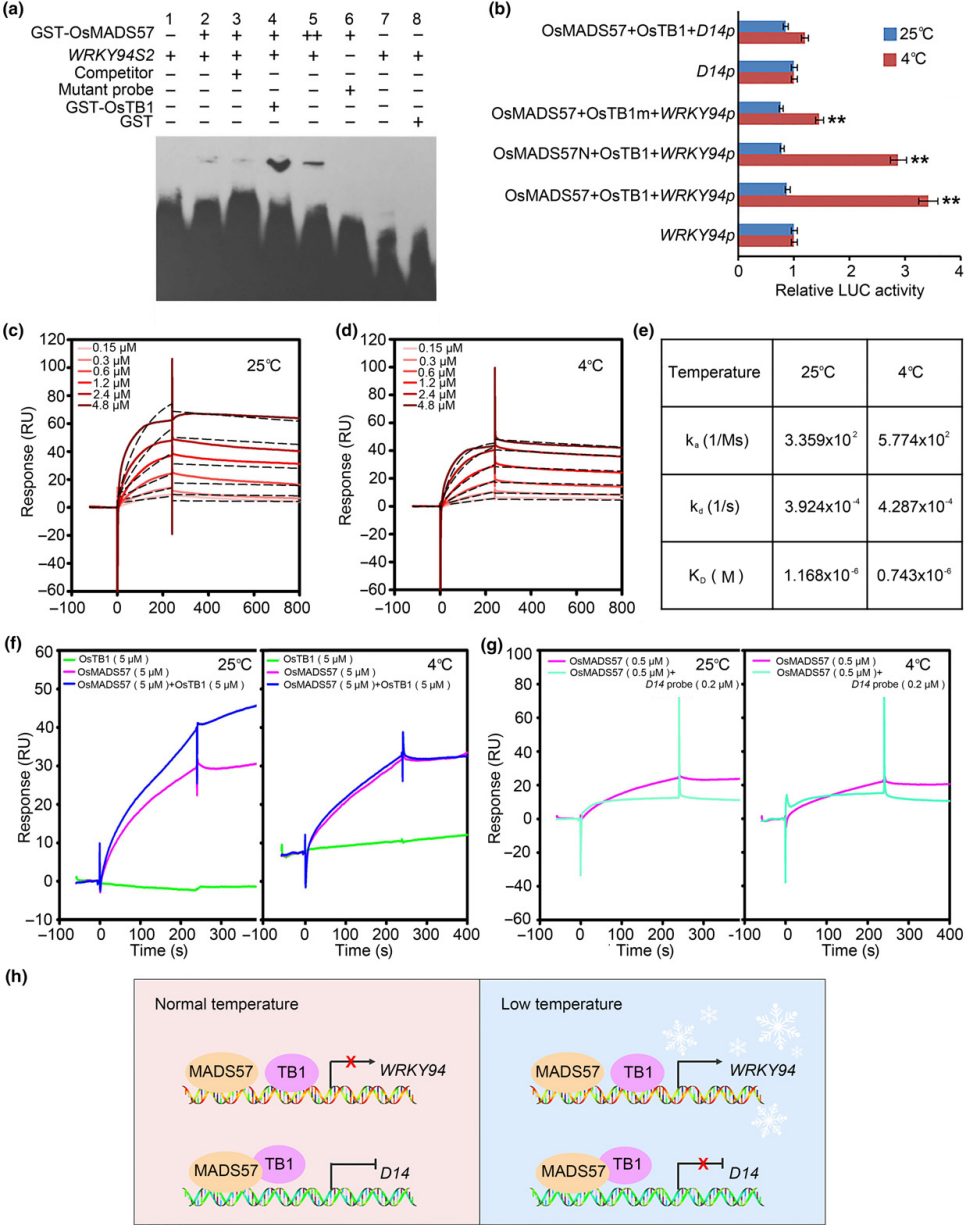
OsMADS57 interacts with OsTB1 to coordinate rice growth and chilling tolerance. (a) Electrophoretic mobility shift assay (EMSA) of the effect of OsTB1 on OsMADS57 binding to the *OsWRKY94* promoter. Excess amounts (×50) of unlabeled oligos were added as the competitors. GST protein served as the negative control. (b) Effect of OsTB1 and OsMADS57 on the transcriptional regulation of *OsWRKY94* in *Arabidopsis* protoplasts. OsTB1m was used as the negative control. **, *P *<* *0.01 (Student's *t*‐test). (c, d) Kinetic binding of OsMADS57 to the *OsWRKY94* promoter subfragment *WRKY94S2* under (c) 25°C and (d) 4°C. The raw data curves are shown in red and the fitted curves in dotted black lines. (e) Kinetic and association constants of OsMADS57 binding to the promoter of *OsWRKY94*; k_a_, association rate constant; k_d_, dissociation rate constant; K_D_, dissociation constant; M, mol l^−1^; s, second. (f) OsTB1 effect on OsMADS57 binding to *WRKY94S2* at 25°C and 4°C. A mixture of 1 μM OsMADS57 and 5 μM OsTB1 was placed in an ice bath for 30 min then passed over the biosensor chip. (g) Assay of *D14* as a competitor for OsMADS57 binding to *WRKY94S2* at 25°C and 4°C. The *D14* probe contained the CATTAAAAAG CArG‐box. (h) Temperature‐dependent regulation of *OsWRKY94* and *D14* by OsMADS57 interacting with OsTB1 to balance rice growth and chilling tolerance. OsMADS57 activates *OsWRKY94* under chilling stress, and represses *D14* under normal conditions.

An EMSA revealed that OsTB1 enhanced OsMADS57 binding to the promoter of *OsWRKY94* (Fig. 5a). The expression of *OsWRKY94::LUC* was significantly induced by the co‐transformation of OsMADS57 and OsTB1 (Fig. 5b). The LxxLL motif that facilitates the interaction between various proteins (Heery *et al*., 1997) was mutated in OsTB1; its sequence, VQWLL, which enables its interaction with OsMADS57, was mutated to AQWAA. When OsMADS57 and the mutated version of OsTB1 (OsTB1^**A**159QW**A**162**A**163^) were co‐expressed, the expression activity pattern of *OsWRKY94::LUC* was decreased at low temperatures compared with plants in which the WT versions of both proteins were co‐expressed (Fig. 5b). In contrast, the temperature‐dependent expression pattern of *D14* was significantly independent of the interaction between OsMADS57 and OsTB1 in the same LUC activity system. The co‐localization assay showed that the fluorescence signal for OsMADS57‐mCherry overlapped with that for GFP‐OsTB1 in the nucleus (Fig. S7, Methods S1).These results demonstrate that OsTB1 enhances the binding of OsMADS57 to the promoter of *OsWRKY94,* enhancing its promotional effect on transcription expression.

In order to confirm the temperature dependence of OsMADS57 binding to the promoter of *OsWRKY94*, a surface plasmon resonance (SPR) biosensor technique was used to perform a kinetic assay with different protein concentrations. The purified recombinant OsMADS57 and OsTB1 proteins were checked, and the *WRKY94S2* oligonucleotide probe was immobilized on the SA‐chip (Fig. S8a,b). The relative responses of the binding of OsMADS57 to the promoter of *OsWRKY94* were dependent on temperature changes, and positively correlated to the protein concentration (0.15–4.80 μM) (Fig. 5c,d). The dissociation rate constant *K*_D_ was 1.2 × 10^−6^M at 25°Cand 0.7 × 10^−6 ^M at 4°C (Fig. 5e).Temperature change showed an effect of OsMADS57‐OsTB1 heterodimerization on its binding to the promoter of *OsWRKY94*. When recombinant OsTB1 was added to the reaction of OsMADS57 and *OsWRKY94S2*, the transcription factor had an increased binding affinity compared with OsMADS57 alone at 25°C; however, no significant change of affinity was observed at 4°C (Fig. 5f). The *D14* probe was mixed into the binding reaction to assess whether it affected the OsMADS57 transcription factor binding to the promoter of *OsWRKY94*. The *D14* probe decreased the binding of OsMADS57 to *OsWRKY94S2* at 25°C, whereas at 4°C, the binding response showed a slight change when *D14* was added (Fig. 5g).These suggested that the affinity of OsMADS57 binding to the promoter of *OsWRKY94* was dependent on experiencing chilling temperatures. *D14* as one of the targets competed with *OsWRKY94* for OsMADS57 binding, which was dependent on temperature.Our data indicate that the chilling‐dependent induction of *OsMADS57* expression together with OsTB1 optimizes organ differentiation via *D14* and the chilling response via *OsWRKY94*, thereby determining the tolerance of rice growth to environmental temperature changes (Fig. 5h).

## Discussion

### OsMADS57 maintains the capacity for chilling tolerance via its network of stragolactone signaling pathways

Hormones such as stragolactone modulate differentiation of axillary buds in the axils of the leaf primordia for plant development (Guo *et al*., 2013; Ha *et al*., 2014).OsMADS57 together with OsTB1 modulate regulation of axillary buds via *D14* as a receptor‐mediating signaling pathway (Guo *et al*., 2013; Zuo & Li, 2014). The meristem, including axillary meristem, is a source for organogenesis in plant development, its tolerance capacity is based on developmental regulation networks, such as OsMADS57‐OsTB1‐*D14*. Our data suggest that OsMADS57 might be a pivotal component to coordinate *OsWRKY94*‐mediated stress responses with *D14*‐mediated leaf organogenesis to adapt to cold environments. Decreased *OsMADS57* expression in the *cold1‐1* mutant during chilling (Fig. S9) suggests that OsMADS57 is involved in the COLD1‐RGA1 sensing pathway for cold tolerance. This result may hint that cold signaling via sensors such as COLD1/RGA1 triggers a series of signals that maintain developmental capability. The gain‐of‐function mutant *osmads57‐1* and the *OsMADS57*‐OE line had improved chilling tolerance, whereas the loss‐of‐function mutant *osmads57‐2* and the *OsMADS57‐*AS line were sensitive to chilling (Figs 1b, S4a). Overexpression of *OsMADS57* increased the tiller number by regulating the outgrowth of axillary buds (Guo *et al*., 2013) and exhibited a strong adaptive response to cope with chilling stress. The outgrowth of axillary buds was visibly increased in *osmads57‐1* after the chilling treatment (Fig. S2b, S4c). Meanwhile, the *d14* mutant*,* as well as the double mutant *d14 osmads57‐1*, had improved chilling tolerance (Fig. 1c). It means that the genes involved in the strigolactone network, such as *OsMADS57*,* OsTB1* and *D14*, may influence plant abiotic stresses. The network may well link the phenotypic adaptation of plants to certain growth environments through its action on meristem initiation and differentiation in response to different stimuli. The chilling tolerant line *osmads57‐1* showed newly differentiated leaves and tillers following the chilling treatment. The increased mitotic index in the gain‐of‐function mutant *osmads57‐1* under the chilling treatment indicates that OsMADS57 probably maintains the cell division capability under stress conditions.

### OsMADS57 triggers its temperature‐dependent targets controlling trade‐off between stress responses and plant development

MADS‐box proteins with DNA binding motif CArG box (CC(AT)_6_GG) target a series of genes for their transcription expression(De Bodt *et al*., 2003; de Folter & Angenent, 2006; Gramzow & Theissen, 2010).The genes for development and stress response are involved in the targets of MADS proteins. The *cis*‐element at which OsMADS57 binds the promoter of *OsWRKY94* was found to be identical to the CArG‐box motif previously reported for *D14* (Guo *et al*., 2013), suggesting that OsMADS57 might act to link the organogenesis and stress response networks. Data from the chromatin immunoprecipitation (ChIP), electrophoretic mobility shift (EMSA) and yeast one‐hybrid assays, as well as the phenotype of *oswrky94‐1*, support the hypothesis that *OsWRKY94* is a direct target of OsMADS57 in the stress response. The affinity of OsMADS57 for *OsWRKY94* was dependent on chilling temperatures, as revealed by dissociation rate constant (*K*_D_) changes at cold temperatures (Fig. 5e). The data suggested a change of OsMADS57 activity on OsWRKY94 from transcriptional repression at normal temperature to activation at the chilling temperature. This reveals that OsMADS57 can act as a transcriptional activator or repressor in plant development to enable tolerance to the ambient temperature. The affinity of OsMADS57 to the targets and the transcriptional dual regulation were dependent on temperature change, which might in turn be based on temperature‐dependent protein modification, such as phosphorylation (Molkentin *et al*., 1996; Badodi *et al*., 2015). The diverse functions of MADS‐box proteins are most likely achieved through interaction with other proteins. However, our data suggested that OsTB1 interacts with OsMADS57 to enhance *OsWRKY94* transcription in protoplasts under a chilling treatment. In the gain‐of‐function mutant *osmads57‐1*,* OsWRKY94* expression was increased relative to the wild‐type (WT) at cold temperatures, whereas it was decreased in the loss‐of‐function mutant *osmads57‐2* (Fig. 2e).The *osmads57‐1* mutant and the loss‐of‐function mutant *ostb1* both improved chilling tolerance, whereas the loss‐of‐function mutant *oswrky94‐1* exhibited chilling sensitivity. Alternative transcription factors, such as OsTB1, may form complexes with the target genes and/or OsMADS57 in the different environmental conditions. Homo‐ or heterodimerization of the component of OsMADS57 and OsTB1 might regulate the target genes flexibly and diversely in response to the ever‐changing environment. OsTB1 suppressed *OsWRKY94* expression in the chilling treatment, which might be explained as a fine regulation for defense and development. Moreover, the temperature‐dependent alternative splicing of MADS‐box transcription factors in response to ambient temperature variation may result in different isoforms that could compete to interact with other factors and affect the DNA binding activity (Pose *et al*., 2013). The two transcription factors OsMADS57 and OsTB1 could therefore converge on the *OsWRKY94* promoter to ensure the fine‐tuned control of *OsWRKY94* expression, coordinating chilling tolerance with the outgrowth of axillary buds (Fig. 5h). OsMADS57 may function as a molecular switch in the cross‐talk between endogenous developmental cues and external signals to coordinate plant development with the response to chilling stress.

In nature, all species may be exposed to environmental stresses at some point in their lifecycle. Our findings support a model in which OsMADS57 acts as a key regulator, enabling the plant to adapt to its environment by balancing cell differentiation, cell division and chilling stress tolerance. OsMADS57 and its interaction partner OsTB1 coordinate the regulation of *D14* for organogenesis and *OsWRKY94* for the stress response. Our elucidation of the molecular roles of OsMADS57 in OsMADS57/OsTB1‐*OsWRKY94/D14* signaling provides novel insights into the complicated regulatory network controlling plant developmental responses to chilling environments.

## Author contributions

L.C. and K.C. designed the experiments; L.C. performed experiments, data analysis and wrote the manuscript; Y.Z., S.X. and Z.Z. contributed to assist in performing part of the experiments; Y.X. and J.Z. contributed to assist in analyzing and discussing the data; and K.C. supervised the project and revised the manuscript.

## Supporting information

Please note: Wiley Blackwell are not responsible for the content or functionality of any Supporting Information supplied by the authors. Any queries (other than missing material) should be directed to the *New Phytologist* Central Office.

**Fig. S1** Molecular identification of the *osmads57‐2* mutant and the *OsMADS57* expression patterns under different abiotic stresses.
**Fig. S2 **Developmental responses of *osmads57‐1*,* osmads57‐2,* and the wild‐type Dongjin to the chilling treatment.
**Fig. S3** Flow cytometry assay of cell division in *osmads57‐1*,* osmads57‐2*, and the wild‐type Dongjin following chilling treatment.
**Fig. S4** Chilling response of *OsMADS57* overexpression and antisense lines.
**Fig. S5 **Molecular identification of the *oswrky94‐1* mutant and the phylogenetic analysis of *OsWRKY94*.
**Fig. S6** Expression patterns and regulation activity of *OsWRKY94* and five other *WRKY* genes in the *osmads57* mutant.
**Fig. S7 **Co‐localization of OsMADS57 and OsTB1 in rice protoplasts.
**Fig. S8** Characterization of purified proteins and quantification of their binding to DNA using SPR.
**Fig. S9 **Relative *OsMADS57* expression in the *cold1‐1* mutant treated at 4°C for 1 or 5 h.
**Table S1** List of primers and accession numbers of genes used in this study
**Methods S1** Materials and methods.
