# Supplementary material for: OsMADS57 together with OsTB1 coordinates transcription of its target OsWRKY94 and D14 to switch its organogenesis to defense for cold adaptation in rice
Source: New Phytol. 2018 Jan 24;218(1):219–31. doi: 10.1111/nph.14977 (PMC5873253; doi:10.1111/nph.14977)
Supplement: Supplementary file 1 — Fig. S1 Molecular identification of the osmads57‐2 mutant and the OsMADS57 expression patterns under different abiotic stresses. Fig. S2 Developmental responses of osmads57‐1, osmads57‐2, and the wild‐type Dongjin to the chilling treatment. Fig. S3 Flow cytometry assay of cell division in osmads57‐1, osmads57‐2, and the wild‐type Dongjin following chilling treatment. Fig. S4 Chilling response of OsMADS57 overexpression and antisense lines. Fig. S5 Molecular identification of the oswrky94‐1 mutant and the phylogenetic analysis of OsWRKY94. Fig. S6 Expression patterns and regulation activity of OsWRKY94 and five other WRKY genes in the osmads57 mutant. Fig. S7 Co‐localization of OsMADS57 and OsTB1 in rice protoplasts. Fig. S8 Characterization of purified proteins and quantification of their binding to DNA using SPR. Fig. S9 Relative OsMADS57 expression in the cold1‐1 mutant treated at 4°C for 1 or 5 h. Table S1 List of primers and accession numbers of genes used in this study Methods S1 Materials and methods. [file NPH-218-219-s001.pdf]

New Phytologist Supporting Information

Article title: OsMADS57 together with OsTB1 coordinates transcription of its target *OsWRKY94* and *D14* to switch its organogenesis to defense for cold adaptation in rice

Authors: Liping Chen, Yuan Zhao, Shujuan Xu, Zeyong Zhang, Yunyuan Xu, Jingyu Zhang and Kang Chong

Article acceptance date: 25 November 2017

Methods S1 – Materials and Methods

Subcellular localization

The coding sequence of *OsWRKY94* was inserted into the pBI221 vector for fusion with GFP under the control of the *35S* promoter to generate the *35S::*OsWRKY94-GFP construct. For the protoplast transformation, protoplast cells were isolated from the leaf sheaths of 10-day-old etiolated rice seedlings and transformed using the polyethylene glycol (PEG 4000) method (Bart *et al.*, 2006). GFP fluorescence was visualized using a confocal microscope (Leica TCS SP5; Leica, Mannheim, Germany) equipped with an argon laser (488 nm); H2B-mCherry (an intrinsic nuclear protein) marker was used and visualized at an excitation wavelength of 543 nm. In colocalization assay, DAPI was used to stain the nucleic acids and then visualized at an excitation wavelength of 405 nm, the GFP and mCherry fluorescence was visualized using a confocal microscope (FV1000MPE; OLYMPUS, Tokyo, Japan).

Histological analyses

Seedlings were treated in a 4 ℃ circulating water bath for four days following two weeks of growth in Kimura B nutrient solution (Kato-Noguchi & Ino, 2005). The SAM and the axillary buds were fixed, cut into 2-μm sections, and stained with toluidine blue for analysis using light microscopy (Carl Zeiss, Mannheim, Germany).

Phylogenetic analysis

The phylogenetic analysis of six WRKY protein sequences from *Arabidopsis* and rice was performed using MEGA 7.0 (Kumar *et al.*, 2016), with default settings and using the neighbor-joining method. Bootstrap values (%) of 2,000 replicates are shown at the branching points.

Fig.S1 Molecular identification of the *osmads57-2* mutant and the *OsMADS57* expression patterns under different abiotic stresses.

Fig.S2 Developmental responses of *osmads57-1*, *osmads57-2,* and the wild type Dongjin to the chilling treatment.

Fig.S3 Flow cytometry assay of cell division in *osmads57-1*, *osmads57-2*, and the wild type Dongjin following chilling treatment.

Fig.S4 Chilling response of *OsMADS57* overexpression and antisense lines.

Fig.S5 Molecular identification of the *oswrky94-1* mutant and the phylogenetic analysis of *OsWRKY94*.

Fig.S6 Expression patterns and regulation activity of *OsWRKY94* and five other *WRKY* genes in the *osmads57* mutant.

Fig.S7 Co-localization of OsMADS57 and OsTB1 in rice protoplasts.

Fig.S8 Characterization of purified proteins and quantification of their binding to DNA using SPR.

Fig.S9 Relative *OsMADS57* expression in the *cold1-1* mutant treated at 4 ℃ for one or five hours.

Table S1 List of primers and accession numbers of genes used in this study.

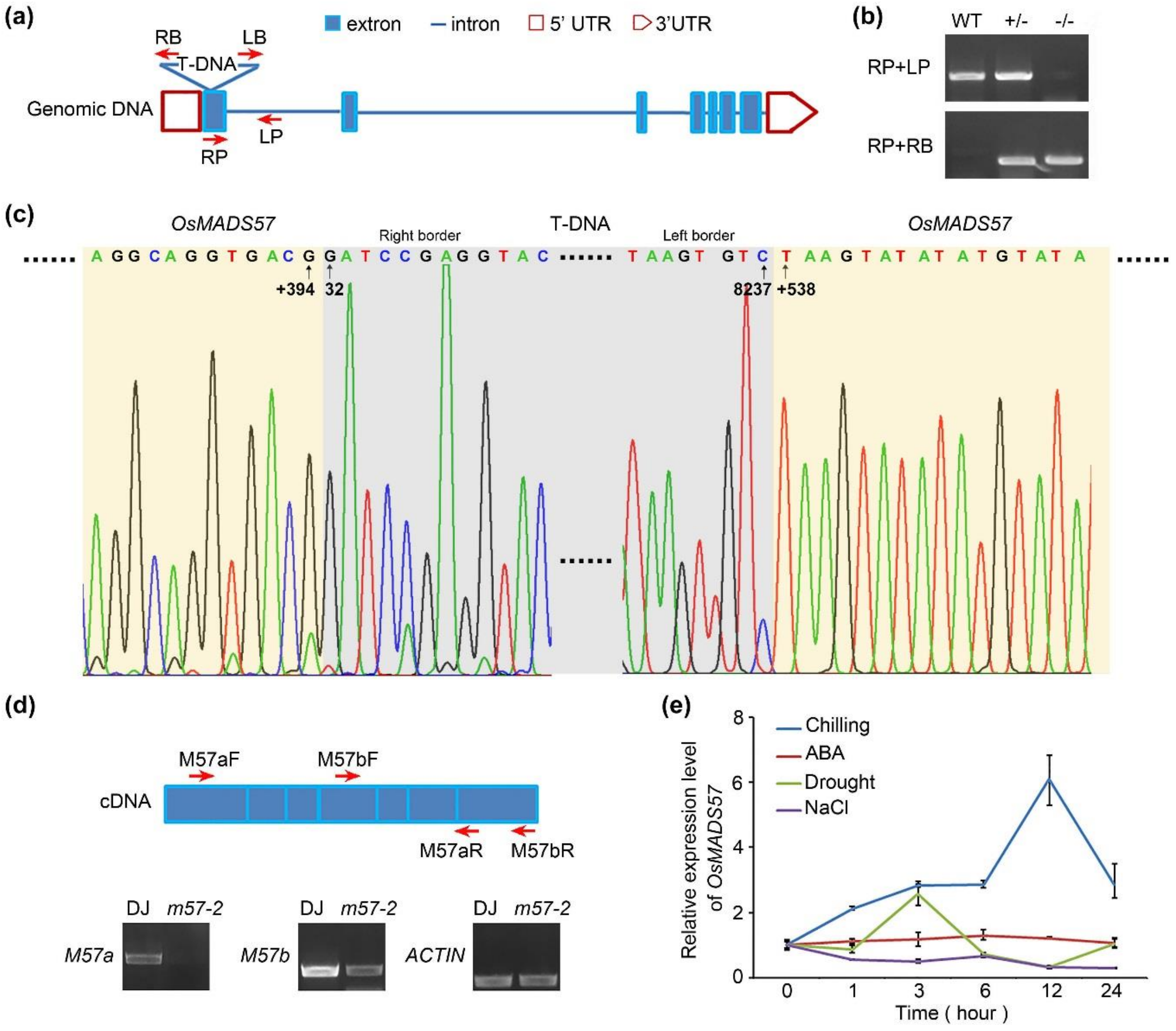

Fig. S1 Molecular identification of the *osmads57-2* mutant and the *OsMADS57* expression patterns under different abiotic stresses.

(a) Schematic of the *OsMADS57* gene. Primers, marked with arrows, are described in Table S1. (b) The homozygous mutant *osmads57-2* (*m57-2*) was identified using PCR. +/−, heterozygous; −/−, homozygous; WT, wild type. (c) Sequence identification of the T-DNA insertion site. The inserted T-DNA sequence is highlighted in gray. (d) RT-PCR expression analysis in various regions of the *OsMADS57* transcript. (e) *OsMADS57* expression patterns under chilling, ABA, drought, and NaCl treatments. One-week-old wild type ZH10 (*Oryza sativa* L. ssp.*japonica* cv. Zhonghua 10) seedlings were treated with 4°C (chilling), 100 μM ABA, 20% PEG6000 (drought), or 200 mM NaCl for specific time courses. Data are means ± SD, n= 3.

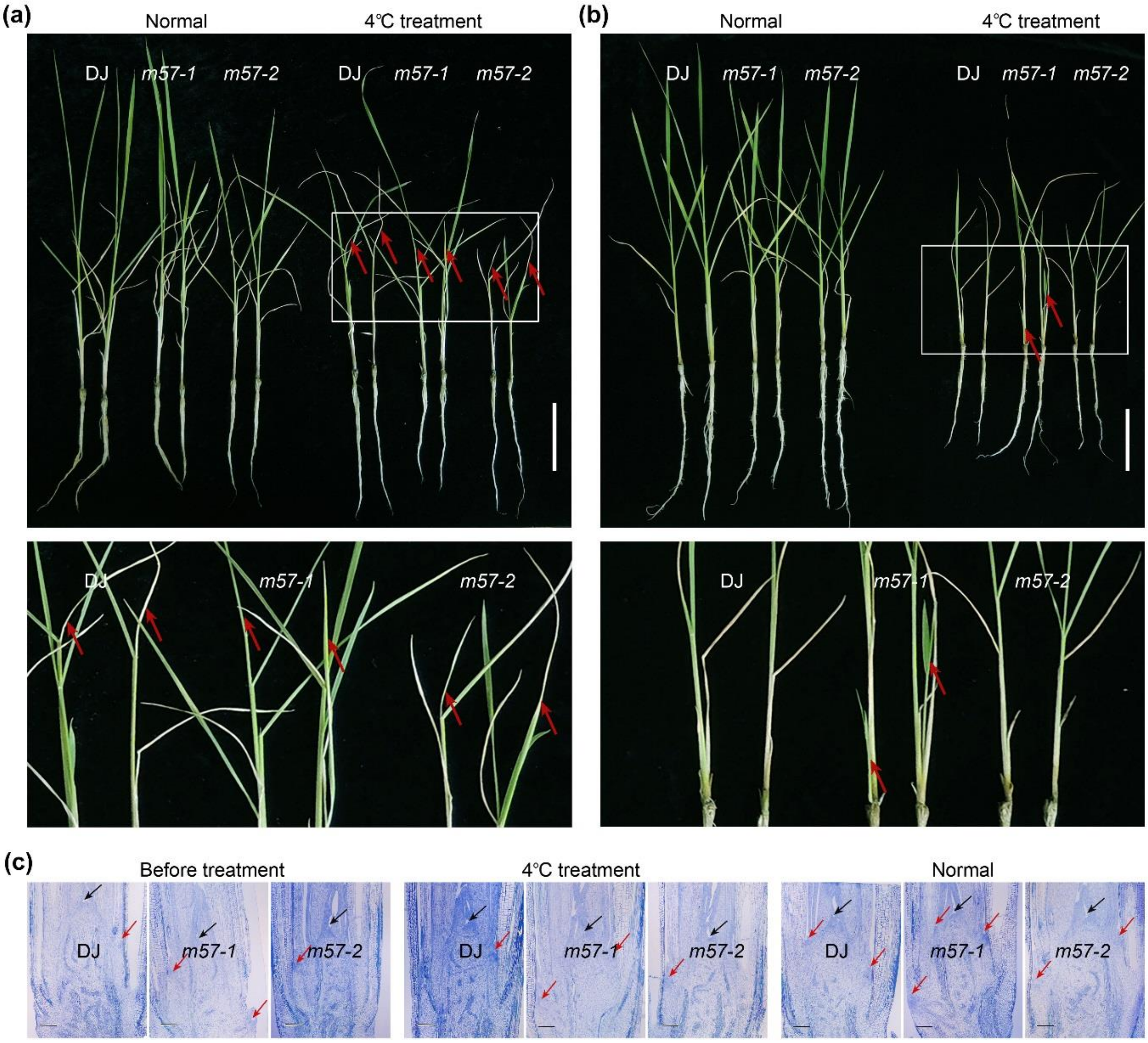

Fig. S2 Developmental responses of *osmads57-1*, *osmads57-2*, and the wild type Dongjin to the chilling treatment.

(a) Phenotypes of the gain-of-function mutant *osmads57-1* (*m57-1*), the loss-of-function mutant *osmads57-2* (*m57-2*), and the wild type Dongjin (DJ, *Oryza sativa* L. ssp.*japonica* cv. Dongjin) after a week's recovery from the chilling treatment. The arrows indicate withered leaves. The boxed areas in the top panels are magnified in the lower panel. Scale bars, 5 cm. (b) Phenotypes of *osmads57-1* (*m57-1*), *osmads57-2* (*m57-2*), and the wild type Dongjin (DJ, *Oryza sativa* L. ssp.*japonica* cv. Dongjin) after more than two weeks' recovery from the chilling treatment. The arrows indicate the newly developed tillers. The boxed areas in the top panel are magnified in the lower panel. Scale bars, 5 cm. (c) Longitudinal sections of the shoot meristems (black arrows) and axillary buds (red arrows). Scale bars, 200 μm.

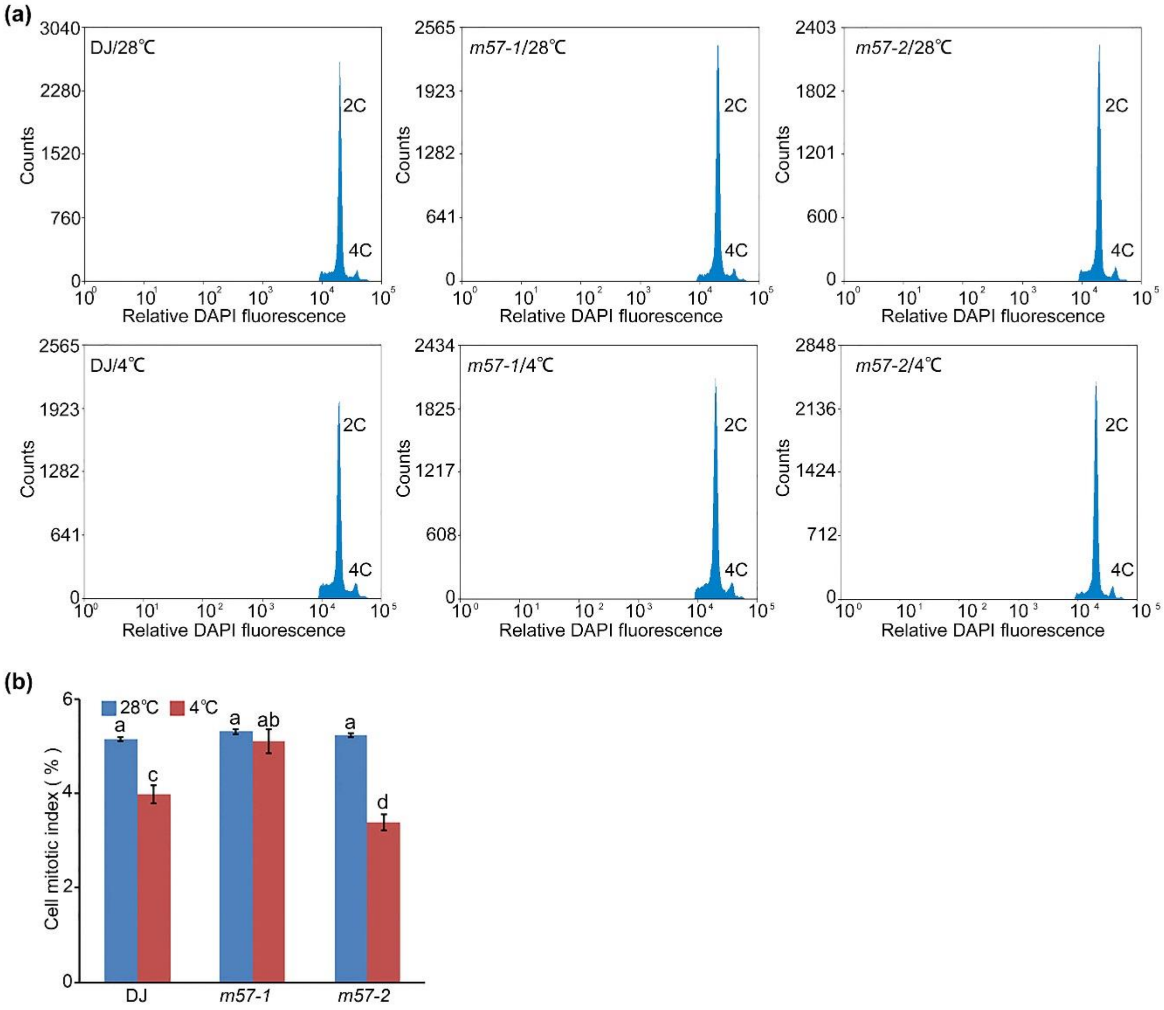

Fig. S3 Flow cytometry assay of cell division in *osmads57-1*, *osmads57-2*, and the wild type Dongjin following chilling treatment.

(a) Seedlings of the gain-of-function mutant *osmads57-1* (*m57-1*), the loss-of-function mutant *osmads57-2* (*m57-2*), and the wild type Dongjin (DJ, *Oryza sativa* L. ssp.*japonica* cv. Dongjin) at 28 ℃ and 4 ℃. Cell nuclei taken from the root apical meristem were stained with DAPI (1 μg/mL) and analyzed using flow cytometry. 2C and 4C represent the DAPI signals that correspond to nuclei with 2C or 4C DNA contents. (b) Cell mitotic index in the root apical meristem. Data represent means ± SD, n =3, the lowercase letters indicate the significance (P<0.05) by one-way ANOVA tests.

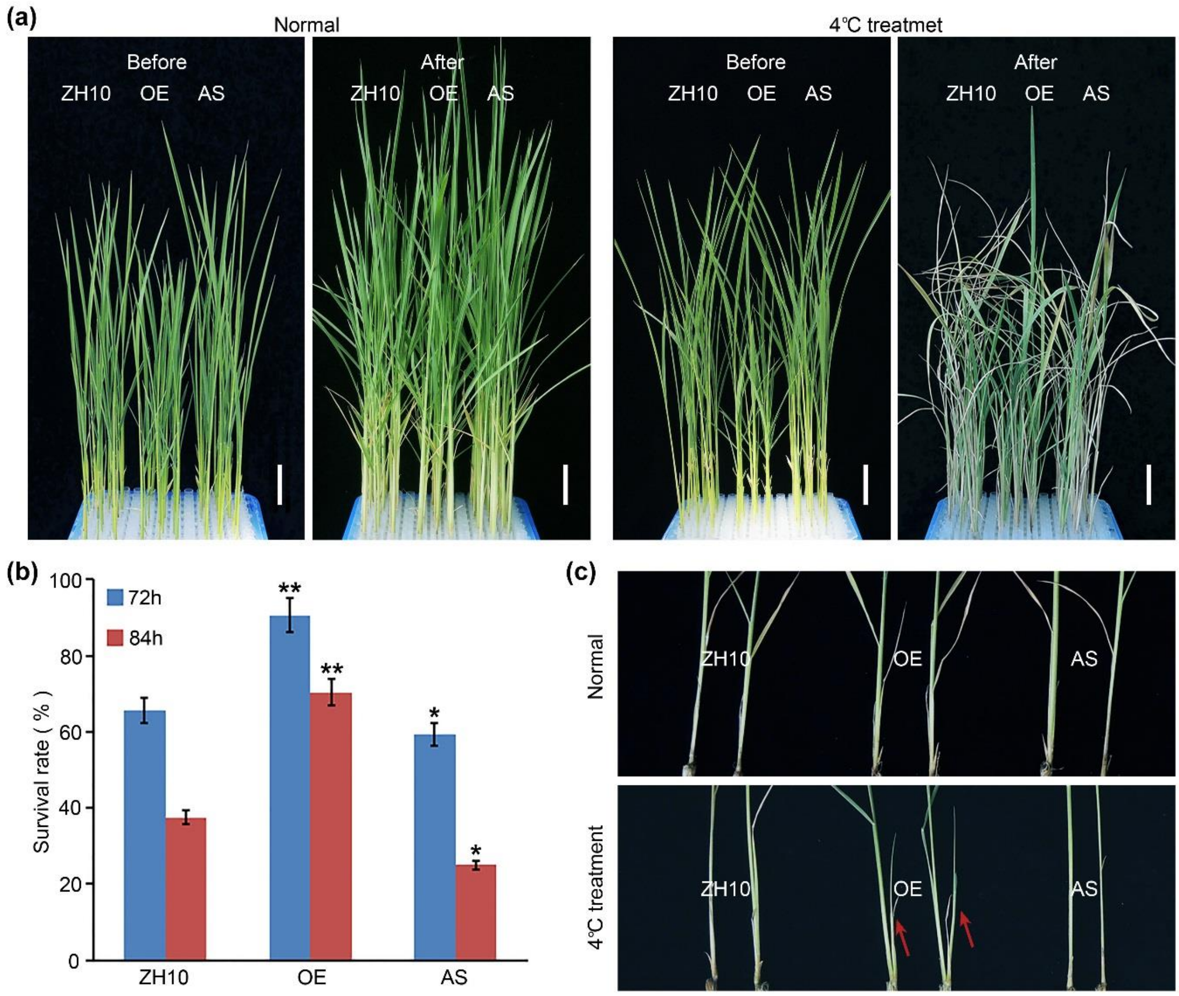

Fig. S4 Chilling response of *OsMADS57* overexpression and antisense lines.

(a) Phenotypes of *OsMADS57* overexpression (OE) line, antisense (AS) line, and the wild type Zhonghua 10 (ZH10, *Oryza sativa* L. ssp.*japonica* cv. Zhonghua 10). Scale bars, 2.5 cm. (b) Survival rate of the *OsMADS57* overexpression (OE) and antisense (AS) lines under chilling treatments of 72 and 84 hours. Data are means ± SD, n = 3, $^{*}P< 0.05$; $^{**}P< 0.01$. Student's t-test. (c) Seedlings from (a) in detail. Chilling treatment increased the tiller growth of the *OsMADS57* overexpression (OE) line. The arrows indicate the newly developed tillers.

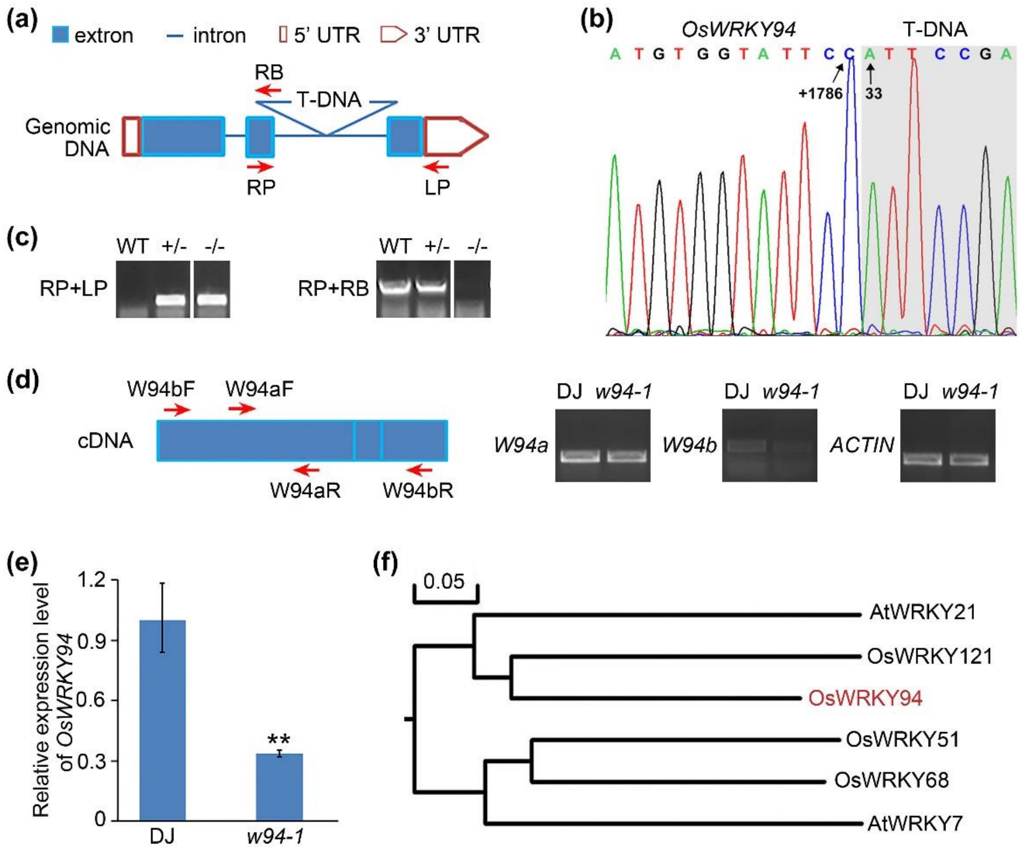

Fig. S5 Molecular identification of the *oswrky94-1* mutant and the phylogenetic analysis of *OsWRKY94*.

(a) Schematic of the *OsWRKY94* gene. Primers, marked with arrows, are described in Table S1. (b) Sequence identification of the T-DNA insertion site. The inserted T-DNA sequence is highlighted in gray. (c) The homozygous mutant *oswrky94-1* (*w94-1*) was identified using PCR. +/−, heterozygous; −/−, homozygous; WT, wild type Dongjin (DJ, *Oryza sativa* L. ssp.*japonica* cv. Dongjin). (d) RT-PCR expression analysis in various regions of the *OsWRKY94* transcript. (e) qRT-PCR analysis of *OsWRKY94* expression in the wild type (DJ) and the *oswrky94-1* (*w94-1*) mutant using primers flanking the T-DNA insertion sites. The expression level in the wild type was defined as 1. Data are means ± SD, n = 3. $^{**}P < 0.01$. Student's t-test. (f) Phylogenetic tree of partial WRKY proteins which showed a high sequence similarity with OsWRKY94 in *Oryza sativa* and *Arabidopsis thaliana.*

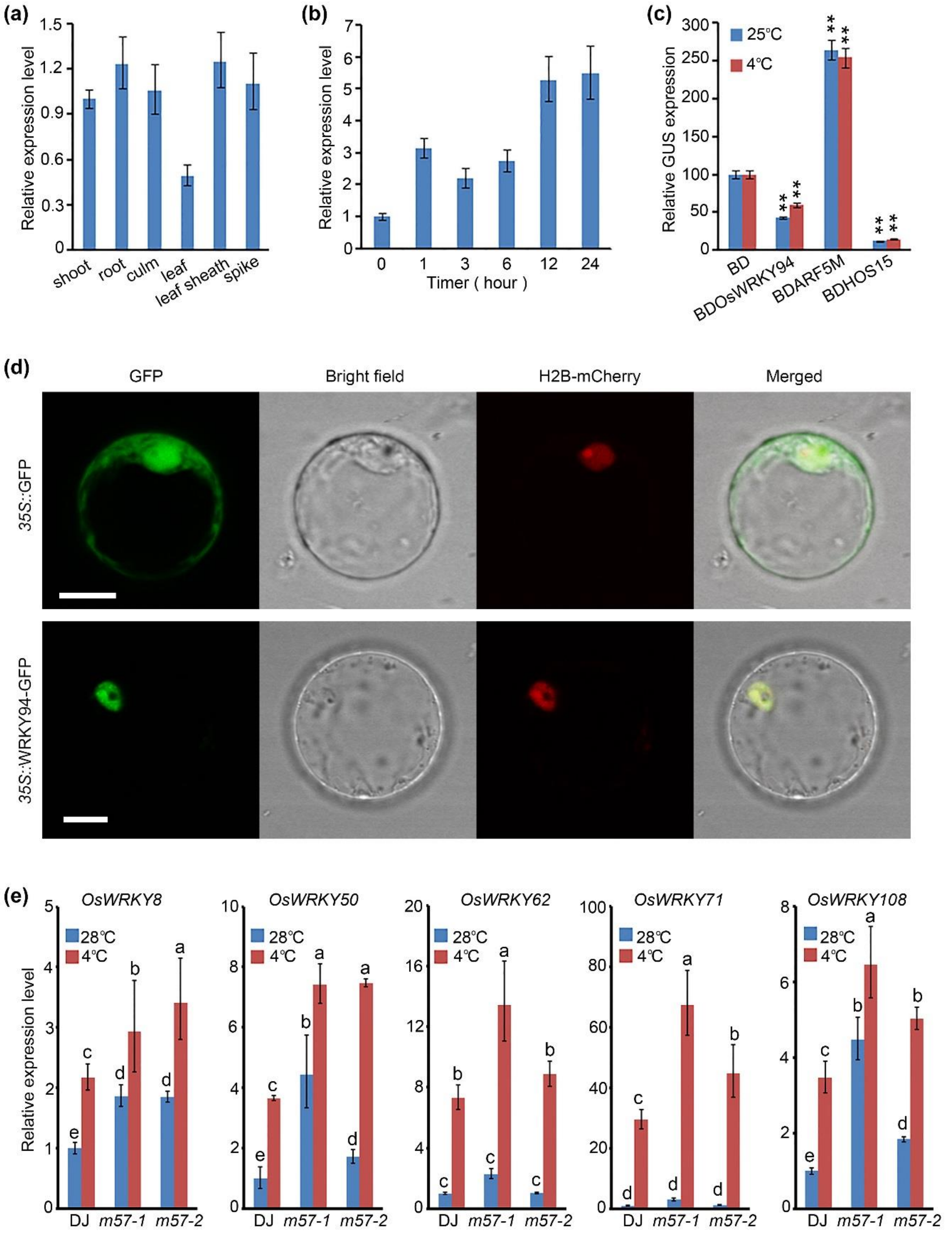

Fig. S6 Expression patterns and regulation activity of *OsWRKY94* and five other *WRKY* genes in the *osmads57* mutant.

(a) *OsWRKY94* expression in the rice tissues of shoot, root, culm, leaf, leaf sheath, and spike. (b) The transcriptional response of *OsWRKY94* to chilling treatments of 1, 3, 6, 12, or 24 hours at 4 ℃. (c) Transcriptional activity assays in *Arabidopsis* protoplasts. HOS15, a transcription suppressor, and

ARF5M, an activator, were used as the controls. $^{**}P < 0.01$. Student's t-test. (d) Localization of OsWRKY94-GFP in riceprotoplasts. OsWRKY94-GFP signal was merged with that of the H2B-mCherry (an intrinsic nuclear protein) marker in rice protoplasts. Scale bars, 10 μm. (e) Expression patterns of *WRKY*s previously found to be differentially expressed in the *osmads57-1* mutant. *UBIQUITIN* was used as a reference gene in the qRT-PCR. The expression levels in the wild type, Dongjin (DJ, *Oryza sativa* L. ssp.*japonica* cv. Dongjin), before the treatment were defined as 1. Data are means ± SD, n = 3, the lowercase letters indicate the significance (P<0.05) by one-way ANOVA tests.

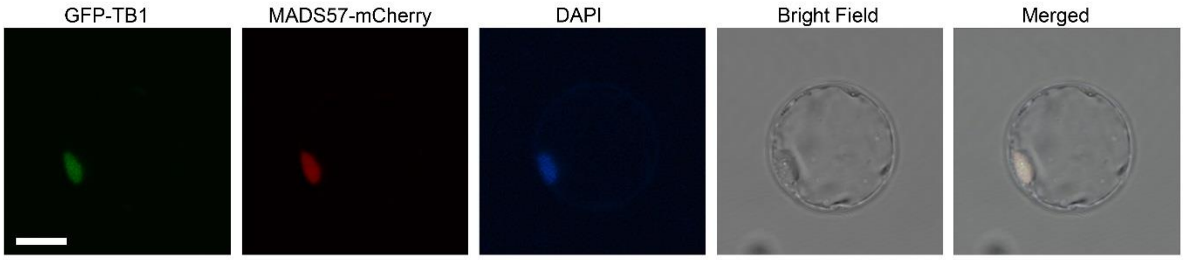

Fig.S7 Co-localization of OsMADS57 and OsTB1 in rice protoplasts.GFP-OsTB1 was co-localizated with OsMADS57-mCherry in rice protoplasts. Scale bars, 10 μm.

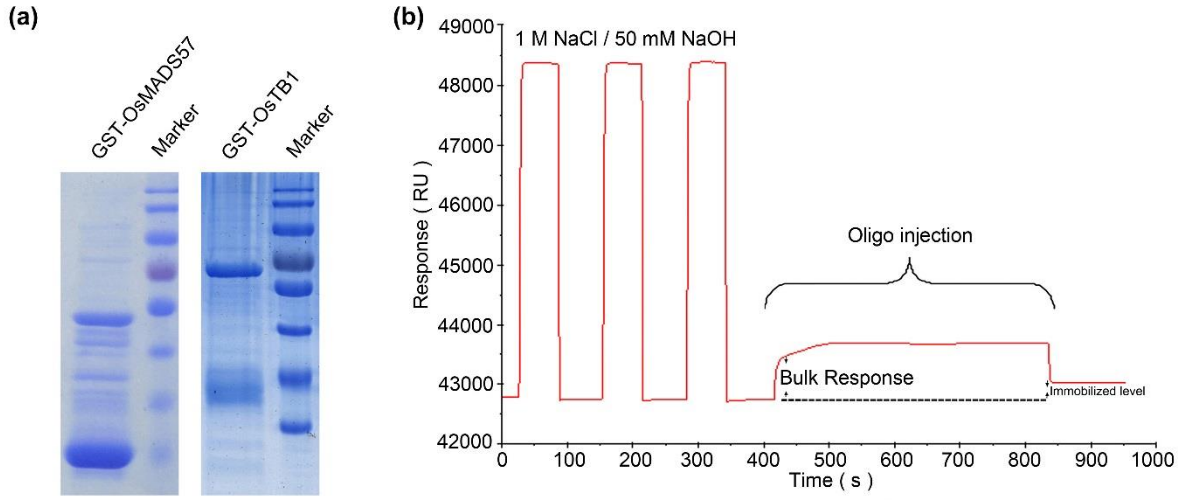

Fig. S8 Characterization of purified proteins and quantification of their binding to DNA using SPR.

(a) SDS-PAGE of purified GST-OsMADS57 and GST-OsTB1 recombinant proteins. A total of 5 μg of each protein was resolved and stained with Coomassie Blue. (b) Tethering of the *WRKY94S2* oligonucleotide probe to the SA-Chip. 251 RU of *WRKY94S2* was immobilized.

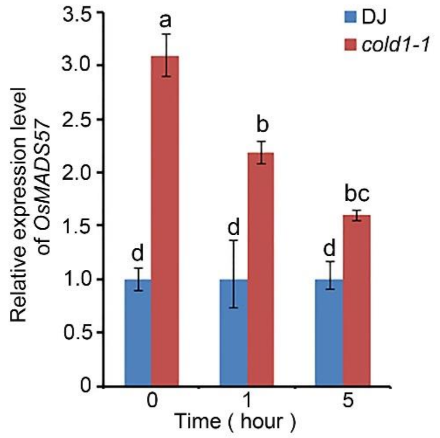

Fig. S9 Relative *OsMADS57* expression in the *cold1-1* mutant treated at 4 ℃ for one or five hours. Data are means ± SD, n =3, the lowercase letters indicate the significance (P<0.05) by one-way ANOVA tests.

Table S1. List of primers and accession numbers of genes used in this study.

Primer name	Primer sequence (5' - 3')
Primers for mutant genotyping	
LB	ACGTCCGCAATGTGTTATTAA
RB	AACGCTGATCAATTCCACAG’
15619LP	TGACTTGGTTGACACGGTTG
15619RP	TTCGCCTTCTCTCCACTCTC
13305LP	CTTGGAGCCTCTGCATTTTC
13305RP	AGGTGCCATTGCTCAAAGAG
Primers for semi-quantitative RT-PCR	
M57aF	GGGGAAGATAGTGATAAGG
M57aR	TTCAAGACTAGGAGGCATA
M57bF	GCAAGAAAGCCACAAGCAAC
M57bR	AAGGCAGATGAAGTCCCAGT
W94aF	TGCTGCCACTCCGTCTACT
W94aR	TGCCACTTCCATCCTCCC

W94bF	GGTGGTGGACATGCTAGAGG
W94bR	TGGCAGCCGAGTATGGTTAT
actinF	GAACTGGTATGGTCAAGGCTG
actinR	ACACGGAGCTCGTTGTAGAAG
Primers for quantitative RT-PCR	
qM57F	CCAGCACCAACATGAAAACTG
qM57R	TGCCTCCCTCTGCCAAATC
qW94F	ATTGCTCAAAGAGGAGGAAGTT
qW94R	CCTTGGATGAGGCGAACC
qUBQF	ACCACTTCGACCGCCACTACT
qUBQR	ACGCCTAAGCCTGCTGGTT
WRKY8PF	AGGATCCGCTTCGTCTTCTT
WRKY8PR	CGTCGGCATATGGTAAACCT
WRKY50PF	CTTCTGCAGGCAGATGATGA
WRKY50PR	CAAGGCACCTTCAATTTGCT
WRKY62PF	GAGACCGTGAAGGATGGGTA
WRKY62PR	AGGTCGCCACTAGCATTGAC
WRKY108PF	ACAGCCTCTCCTTACGACGA
WRKY108PR	GTGTAGACGACGACGTGCAG
WRKY71PF	TCAGCCTGGTGGTGAAAGAT
WRKY71PR	ACGAGGATCGTGTTGTCCTC
Primers for yeast one hybrid	
M57*Eco*RI	GGAATTCATGGGGAGGGGGAAGATAGTGA
M57*Xho*I	CCCTCGAGTAAGGCAGATGAAGTCCCAGTT
TB1*Eco*RIF	CCGGAATTCATGCTTCCTTTCTTCGATT
TB1*Xho*IR	CCGCTCGAGTCAGCAGTAGTGCCGCGAA
W94p*Kpn*IF	CGGGGTACCGGTCCCGTTTGGTAAGGT
W94p*Sal*IR	ACGCGTCGACCGCCGCCGCCGCCGCCGCCGCC
W94pdelt*Kpn*IF	CGGGGTACCAGTTAGATTTAGGGTGAAGATGTG
W94pdelt*Sal*IR	ACGCGTCGACCGCCGCCGCCGCCGCCGCCGCC
D14p*Eco*RI	CGGAATTCACCCGGTGCTTTGTTATG
D14p*Kpn*I	GGGGTACCTGACTCTTGCCTCGCTTC

Primers for EMSA

M57*Eco*RIF	GGAATTCATGGGGAGGGGGAAGATAGT
M57*Bam*HIR	CGGATCCTTAAGGCAGATGAAGTCCCAGT
TB1*Eco*RI	CGGAATTCATGCTTCCTTTCTTCGATTC
TB1*Bam*HI	CGGGATCCTCAGCAGTAGTGCCGCGAAT
W94S2F	TTAACTTTAAAAAGTTAG
W94S2R	CTAACTTTTTAAAGTTAA
57mPF	TTAAAGGGCCCCCTTTAG
57mPR	CTAAAGGGGGCCCTTTAA
TB1P1F	AGAGTGGTCCGCTA
TB1P1R	TAGCGGACCACTCT
TB1P2F	ACGATGGGCCGCCG
TB1P2R	CGGCGGCCCATCGT
TB1mPF	ACGAAAAAAAGCCG
TB1mPR	CGGCTTTTTTTCGT

Primers for subcellular localization and co-localization

221W94PF	ACGGGGGACTCTAGAATGGAGGAGGAGGTGGA
221W94PR	TTTACCCATGGTACCGGTCTGGGCTGACTGTG
GFP-TB1PF	GGAGGTGGACCCTCTAGAATGCTTCCTTTCTTCGATTCCC C
GFP-TB1PR	TGTTTGAACGATCGGTACCGCAGTAGTGCCGCGAATTG
MADS57mCherryPF	GGGGTACCATGGGGAGGGGGAGATAGTGA
MADS57mCherryPR	CGGGATCCAGGCAGATGAAGTCCCAGTT

Primers for transcription activity assay

M57*Xho*IF	CCGCTCGAGATGGGGAGGGGGAAGATAGT
M57*Kpn*IR	GGGGTACCAGGCAGATGAAGTCCCAGT
M57N*Xho*IF	CCGCTCGAGATGGGGAGGGGGAAGATAG
M57N*Kpn*IR	CGGGGTACCATTTAGGCTTCTAGAAAGTTCG
TB1*Xba*IF	GCTCTAGAATGCTTCCTTTCTTCGATTC
TB1*Kpn*IR	GGGGTACCGCAGTAGTGCCGCGAATTG
W94p*Pst*I	AACTGCAGGGTCCCGTTTGGTAAGGT
W94p*Bam*HI	CGGGATCCCGCCGCCGCCGCCGCCGCCGCC

W94pdelt*Pst*IF	AACTGCAGTTAGATTTAGGGTGAAGATGTG
W94pdelt*Bam*HIR	CGGGATCCCGCCGCCGCCGCCGCCGCC
D14p*Pst*IF	AACTGCAGACCCGGTGCTTTGTTATG
D14p*Bam*HIR	CGGGATCCTGACTCTTGCCTCGCTTC
W94*Sma*IF	TCCCCCGGGATGGAGGAGGAGGTGGAGGC
W94*Sal*IR	ACGCGTCGACTCACATGACCGGTCCGACCG
Primers for ChIP	
FLAG57F	CGGGATCCATGGATTACAAGGATGACGACGATAAGATG GG
	GAGGGGGAAGATAGT
FLAG57R	GGGGTACCTTAAGGCAGATGAAGTCCCAGT
UBQ5ChIPF	TATCCAACATGAATGCCACA
UBQ5ChIPR	CAGCACGAGATGAGTAAAACAA
W94S1F	CACAACATACAACTTAATGACCTCAC
W94S1R	TGTGGGCAATGATAGAAAGTCT
W94S2F	GCAAAGACCCAGAGTTTGGT
W94S2R	CCCACATCTTCACCCTAAATC
W94S3F	AGCCGAGCTCCAAAGCTA
W94S3R	CGGAGTTGGGAGAGGAGTAG
W94S4F	GATTTGATGGCTGGTTGCT
W94S4R	GCACACGGAGAAGAAGACAA

Accession numbers	
OsMADS57	Os02g49840
OsTB1	Os03g49880
OsWRKY94	Os12g40570
D14	Os03g10620
OsWRKY8	Os05g50610
OsWRKY50	Os11g02540
OsWRKY62	Os09g25070
OsWRKY71	Os02g08440
OsWRKY108	Os01g60600
COLD1	Os04g51180

References

Bart R, Chern M, Park CJ, Bartley L, Ronald PC. 2006. A novel system for gene silencing using siRNAs in rice leaf and stem-derived protoplasts. *Plant Methods***2**: 13.

Kato-Noguchi H, Ino T. 2005. Possible involvement of momilactone B in rice allelopathy. *Journal of Plant Physiology***162**: 718-721.

Kumar S, Stecher G, Tamura K. 2016. MEGA7: molecular evolutionary genetics analysis version 7.0 for bigger datasets. *Molecular Biology Evolution***33**: 1870-1874.
